# Recurrence of Congenital Heart Disease in Cases with Familial Risk Screened Prenatally by Echocardiography

**DOI:** 10.1155/2011/368067

**Published:** 2011-10-01

**Authors:** Vlasta Fesslova, Jelena Brankovic, Faustina Lalatta, Laura Villa, Valerio Meli, Luciane Piazza, Cristian Ricci

**Affiliations:** ^1^Department of Pediatric Cardiosurgery, Center of Fetal Cardiology, Policlinico San Donato IRCCS, Via Morandi 30, San Donato Milanese, 20097 Milan, Italy; ^2^Clinical Genetic Unit, Fondazione IRCCS Cà Granda Ospedale Maggiore Policlinico, 20122 Milan, Italy; ^3^Obstetrics and Gynecology Unit, Ospedale San Giuseppe, 20123 Milan, Italy; ^4^Neonatal Intensive Care Unit, Ospedale B. Romeo, 98066 Patti, Messina, Italy; ^5^Clinical Epidemiology and Biometry Unit, Policlinico San Donato IRCCS, 20097 Milan, Italy

## Abstract

*Objectives*. To evaluate the recurrence of congenital heart disease (CHD) in pregnant women with familial risk who had been referred for fetal echocardiography. *Material and Methods*. 1634 pregnancies from 1483 women with familial history of CHD in one or more relatives were studied. Fetal cardiologic diagnosis was compared with postnatal findings at 6 months or at autopsy. *Results*. Total recurrence rate of CHD was 3.98%, 4.06% in single familial risk, 2.9% in double, and 5% in multiple risk. It was 3.5% in case of one previously affected child; 4.5% with 2 children; 5.2% with the mother alone affected and 7,5% with father alone affected and 3.5% with a single distant relative. Exact concordance of CHD was found in 21.5% and a partial concordance in 20% of cases. *Conclusions*. Our data show a higher recurrence rate of CHD than previously published data and high relative risk ratios compared to normal population.

## 1. Introduction

The inheritance of congenital heart disease (CHD) has been analyzed in familial studies undertaken by Nora et al. [1–6] and in studies on twins and siblings [7, 8] and the polygenic-multifactorial model for inheritance has been generally accepted. Thus, the incidence of CHD in first-degree relatives was predicted to be between 1 and 5%. However, the polygenic mode of inheritance seemed to be partly contradicted by a higher recurrence risk (16%) for offspring of patients with CHD as reported by Whittemore et al. [9] or a 9% recurrence reported in parents with specific defects [10]. It should be noted that the above are older data obtained in the era before echocardiography. while nowadays our detection of even mild forms of CHD have significantly improved.

A family history of CHD is a frequent reason for referring a patient for fetal echocardiography. Prenatal counseling in these patients is very difficult, because it is not always possible to exclude smaller or evolutive lesions in examined fetuses and the final prognosis of the affected cases may be sometimes completed only after birth. The recurrence rate in populations with familial risk referred for fetal echocardiography was first reported by Allan et al. [11] and then in two other studies [12, 13]. In 1998, our group analyzed retrospectively a small series of fetal cases from families with a history of CHD [14]. We present now a prospective study on a much larger number of cases, extending the followup to at least 6 months, in order to detect even milder lesions. 

## 2. Patients and Methods

### 2.1. Patients

1634 consecutive pregnancies from 1483 women referred for familial history of CHD were studied in our center by fetal echocardiography between January 1995 and June 2010. Patients' families pedigree was collected for at least three generations and the patients were informed about the limits of the study. 

The familial case with CHD who prompted the referral was defined as the index case: it was either an affected mother or father, or a sibling of the current pregnancy, or more distant relatives of the 2nd-3rd degree; 1477 cases were found to have a single familiar risk while 157 cases had multiple risks (2–5 relatives with CHD). One hundred thirty-nine women were examined in two, 12 in three and one in four consecutive, pregnancies, respectively. There were 21 twin pregnancies. 

#### 2.1.1. Inclusion/Exclusion Criteria

Only cases with a known followup were included while mothers with pregestational diabetes or those taking drugs during pregnancy were excluded, as were the index cases affected with Mendelian diagnosed syndromes or chromosomal anomalies, or cases of consanguineous parents.

#### 2.1.2. Methods

Echocardiographic studies were performed using echocardiographic machines Acuson Sequoia 512, Siemens, Erlangen, Germany and Vivid 7, General Electrics, Healthcare Italy or Aloka Prosound Alpha 10, Tokyo, Japan, applying standard methodology with use of 2D, Doppler and Color Doppler techniques. These equipments had all a very good imaging standard. Studies were routinely recorded onto videotape or by digital storage of the echocardiographic machines. All studies were performed by one single experienced fetal cardiologist (V. Fesslova) and discussed with the team of perinatal medicine*.*

Fetal cardiologic diagnosis of the cases was compared with the findings at autopsy, or postnatally, at follow-up examinations at 6 months of age or later. Regarding the differential diagnosis between a physiological patent foramen ovale and ASD secundum, we considered as a criterion the diameter >8 mm in the third trimester and >5 mm at 1 year of age.

All live-born infants either underwent a cardiologic examination in our centre or, if carried out elsewhere, the parents forwarded to us a follow-up questionnaire given to them at the last fetal examination, completed by the team that performed the examination. If necessary, the parents or cardiologists of other centres were contacted by one of our group in order to have a correct postnatal diagnosis.

#### 2.1.3. Analysis of the Data

The data regarding the index case and the echocardiographic findings of the examined fetus, with his subsequent outcomes, were analyzed by means of a computerized database (Filemaker Pro, VI, Claris Corp., Santa Clara, Calif, USA).

Cardiac defects were classified using the sequential segmental classification described by Tynan et al. [15]. We also included the cases in which there was an uncertainty about a precise cardiac diagnosis of the index case, in order to assess the global recurrence of the population referred for familial history.

Defects of the index case and of the examined fetus were analyzed for concordance and discordance. The defect in the examined fetus was described as exactly concordant if it was identical to that one seen in the index case and concordant for the group if the defect belonged to the same spectrum of CHD as seen in the index case, that is, group of shunts, conotruncal lesions, left and right heart obstructive lesions, and laterality defects/dextrocardia, according to the criteria used in previous studies [13, 16].

The *recurrence rate* of CHD was analyzed by subdividing the population into categories of a single- or multiple-familial risk and according to the type of CHD of the index case (Tables 1 and 2), the recurrence rate being defined as a percentage of affected fetuses for the category of familial risk or for the type of CHD in the index case.

*Relative recurrence risk ratios (RRs) *with respect to the normal population were estimated by means of logistic linear regression performed by SAS Software Package version 9.1.3. comparing the frequency of single types of CHD recurrent in our population to the figures of prevalence of similar defects in a study of Moons et al. [17] found in the population of 111.225 normal infants born in Belgium in the year 2002 (with a total prevalence of CHD of 8.3/1000). This study was found to fit better for a comparison with our population, because it regards equally a Caucasian population and, mainly, has a high number of normal infants born within the same year, with a detailed prevalence of single-cardiac lesions.

## 3. Results

### 3.1. Cardiac Lesions Found in Examined Fetuses

**Figure 1:**
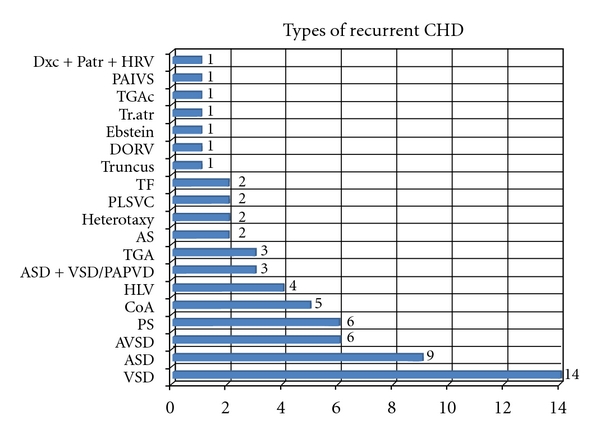
Types of recurrent CHD. Dxc + Patr + HRV: dextrocardia + pulmonary atresia + hypoplastic right ventricle, PAIV: pulmonary atresia and intact septum, TGA-TGAc: transposition of great arteries, corrected transposition of great arteries, DORV: double outlet right ventricle, TF: Tetralogy of Fallot, PLSVC: persistent left superior vena cava, AS: aortic stenosis, ASD: atrial septal defect, VSD: ventricular septal defect, PAPVD: partial anomalous pulmonary venous drainage, HLV: hypoplastic left ventricle, CoA: coarctation of aorta, PS: pulmonary stenosis; AVSD: atrioventricular septal defect.

CHD was found in 65 infants with 2 being twins.  Figure 1 shows the distribution of single types of lesions: more common were septal defects: VSD (14 cases, 21.5% of the defects), ASD (8 cases, 12.3%), and AVSD and PS (in 6 cases, resp.).

Twelve cases (18.4%) were diagnosed only after birth: one case with coarctation of the aorta who was examined in utero only in the 2nd trimester, 7 small VSDs, and 4 ASDs—confirmed at 12 months of age, after the fetal findings in the third trimester of a large foramen ovale (greater than 8 mm) and a large aneurysm of the foramen.

### 3.2. Total Recurrence Rate in Single Categories of Risk

Total occurrence of CHD was 65/1634 (3,98%), 60/1477 (4.06%) in cases with single familiar risk, 2.9% (4/137) and 5% (1/20) in pregnancies having, respectively, double and multiple risk (Table 1).

When excluding the cases that were not diagnosed in utero but found only after birth, the recurrence rate would be lower—of 3.2% (53/1634). As we stated in the Patients and Methods Section, the detailed analysis is based upon the total number of CHD in infants at 6 months of age.

The recurrence rate of CHD was 3.5% (29/818) when one previous child was affected, but increased to 4.5% (1/22) when 2 previous children had CHD.

When mother alone was affected, the recurrence was 5.2% (13/250); with additional 1-2 relatives the recurrence rate increased to 9.7% (3/31).

When father alone was affected, the recurrence rate was 7.5% (7/93) and 4.5% (1/22) with 1-2 other relatives affected. In cases of a single 2nd-3^rd^-degree relative affected, the recurrence was 3.5% (11/316).

### 3.3. Recurrence in Subsequent Pregnancies

Five fetuses of the 139 women who were followed in 2 pregnancies were affected (3.6%), with concordant recurrence of ASD in 2 infants of mothers affected with the same lesion; another mother with VSD had an infant with ASD and two siblings of index cases with TGA and AVSD had both a VSD.

Twelve women who were followed in their 3rd pregnancies had all normal infants: 2 were affected mothers and one affected father who have each had an affected child previously; 2 other affected mothers had normal children in both previous pregnancies, while in the remaining pregnancies normal infants were born after one previously affected child.

One woman, followed up in 4 pregnancies, after the first child with TGA and who had another distant relative affected with an undefined CHD, delivered normal offspring in the subsequent pregnancies.

Table 2 shows in detail the total recurrence rates in the various subgroups of CHD present in the index cases, distinguishing the total rates, and those in siblings, offspring, and distant relatives. It is evident that the recurrence rates of different types of CHD varied between the categories of index cases (i.e., recurrence rate in AVSD in siblings is 6.5% and in affected mothers increased to 1/4 (25%)). The recurrence rate was highest in cases of pulmonary atresia and intact septum (2/14 = 14.3%, with cases affected with pulmonary stenosis), in cases of Ebstein/non-Ebstein anomaly (2/21 = 9.5%). A high recurrence rate of 8.7% (2/23) was also found in the index cases with dextrocardia, with a concordant recurrent anomaly in one fetus (hypoplastic right ventricle and pulmonary atresia), while the second fetus, of an index case with dextrocardia, corrected TGA and pulmonary atresia, had right isomerism and atrioventricular septal defect. Corrected TGA and pulmonary atresia with VSD occurred at a rate of 1/14 = 6.7% and 1/19 = 5.3%, resp.). Surprisingly, severe anomalies such as HLV and univentricular heart had in our series a low recurrence rate of 1.25% and 1.5%, while Fallot's Tetralogy recurred at 6% rate.

*Concordance between index cases and affected fetuses* is summarized in Table S1 (available as a supplementary file).

*Complete exact concordance* was found in 14/65 cases (21.2%).

*Partial concordance* or concordance within group was observed in 13/65 (20%). When the mother alone was affected, an exact concordance occurred in 3/13 cases (23.1%) and in case of a mother and other 3 relatives affected; a partial concordance occurred in further index cases of 5 mothers and in one case when mother and a previous sibling were affected.

When the father alone was affected, an exact concordance occurred in 1/7 cases and a partial one in another case.

When one previous child was affected, the complete concordance was 8/29 (27.6%) and a partial one in 2/29 (6.9%).

In 34 cases (51.5%), there was a *discordance *in the type of cardiac lesion.

1. Lesions of a similar gravity in 5 cases.
2. Milder lesions in 19 cases.
3. More complex CHD in 10 cases.

The type of recurrence could not be defined in 4 cases because of a nonspecific diagnosis of the cardiac anomaly in the index case.

### 3.4. Multiple Familiar Risk

Out of 5 affected fetuses with double/multiple familiar risk there was one case with *an exact concordance* of incomplete AVSD in 3 generations—mother, grandfather, and examined fetus, while two other relatives of the same family had a complex form of AVSD with coarctation of the aorta and another, a more distant relative had a valvular aortic stenosis.

Table 3 shows the relative risk ratios for specific cardiac defects as compared to the data of Moons et al. [17], generally ours are much higher with respect to the risks of a normal population, mainly as for VSD, Tetralogy of Falot and pulmonary atresia with intact septum.

## 4. Discussion

Epidemiology and genetics of cardiac malformations have been an object of several studies over several last decades. The large epidemiological Baltimore Washington Infants Study [18, 19] that analyzed a cohort of children with CHD from 1981 to 1989 has shown also that 30% of affected cases were associated with other genetic disorders. In the population of nonsyndromic children, there was a recurrence of 4.9% in the first degree relatives and the recurrence rate was particularly high in families with HLV and coarctation of the aorta (13 and 8%, resp.). 

Nora [1] introduced in 1968 the hypothesis of a multifactorial mode of inheritance of CHD in the majority of cases, suggesting that several loci could interact in association with environmental factors. They reported a recurrence rate of about 3%, for healthy nonconsanguineous parents with one child affected increasing up to about 10%, after having another affected child; but the risk was variable for different types of CHD. 

Subsequent studies of Burn et al. [7, 8] provided counseling figures for different types of cardiac lesions in siblings and offspring of cases with CHD, reporting a higher recurrence rate for children of affected mothers compared to the cases with an affected father (4.1% and 2.2%, resp.). 

Higher recurrence rates in the offspring of mothers with CHD with and without surgical treatment (of 16.1%) were reported by Whittemore et al. [9], while Rose et al. [10] found a 9% recurrence in parents with specific defects. 

A positive family history of CHD is a frequent cause for the referral for prenatal cardiac investigation which led to the first study of the recurrence of CHD in the population of mothers referred for fetal echocardiography for a family history [13]. In this study, the authors reported a total recurrence rate of 1/52 (1.9%) after one previously affected child and 1/10 after two previously affected children. In a more recent large study in a similar population, Tynan et al. [15] reported a recurrence rate of 2.7%, in fetuses with one or more first-degree relatives with CHD.

The present study included also the cases studied by fetal echocardiography because of a family history of CHD in either first degree or more distant relatives. We insisted on a final cardiologic examination of the child at least at 6 months of age or later, in order to detect even those smaller anomalies, that are difficult to visualize in the fetus [20]. Our results demonstrate a higher recurrence rate for CHD than do the above two fetal studies, with a total recurrence rate of 3.98%, a rate of 4.06% in cases with a single familial risk, of 2.9% in cases with double risk and 5% (1/20) in those with multiple risks. 

The recurrence rate would have been lower if we have had considered only cases diagnosed during fetal life (3.2%), more similar to the results of Gill et al. [13]. We felt it essential that we should consider as recurrent even the milder congenital heart lesions, such as small VSDs, discovered after birth that are often not detectable by fetal echocardiography as well as ASD that cannot usually be seen in the fetus, unless they are truly large. The occurrence of these defects are important for the family that is usually very anxious after a previous experience with a cardiac problem and also for our own knowledge of the recurrence. 

As for the single categories of familial risk, the recurrence rate in our study was 3.8% (30/784) when one previous child was affected increasing to 4.8% (1/21) with 2 previous children with CHD, in agreement with several other reports [2, 8, 12].

Our recurrence rates for siblings and offspring correspond to those reported by Nora and Nora [3] and Rose et al. [10] and others based upon the postnatal data, but they are lower than the those of Whittemore et al. [9] who reported a very high incidence rate (60/372–16.1%) in live-born infants of mothers with and without surgical treatment of CHD. 

We found a difference between the recurrences in offspring of affected mothers and fathers. The preponderance of affected offsprings of mothers with CHD may be at least partly explained by cytoplasmatic inheritance, that is, transmission of maternal cytopathy, while the paternal transmission is of about 2% lower than the maternal one [6, 8, 9]. This mechanism is more obvious in some specific maternal cardiac lesions such as AS, CoA, PS, VSD, and AVSD. On the whole, mothers with CHD are very likely to be more vulnerable to teratogens than fathers [8].

*Frequency of CHD found among our affected fetuses* corresponds to the figures reported in a large epidemiological study of Moons et al. [17] that indicated a global prevalence of CHD in Belgium in the 2002 of 8.3/1000. Another recent study in Sweden, in contrast, reported a much lower prevalence of 2.8/1000 [21]. These differences show how difficult it is to determine the correct prevalence and relative risk for CHD, very likely due to different assessment policies. 

*Recurrence risk ratios *of our series with familial history of CHD show high figures in comparison to the data of the study of Moons et al. [17] regarding the normal population, mainly for VSD, Tetralogy of Fallot, and pulmonary atresia with intact septum. 

High relative risk recurrence ratios both of a specific and discordant CHD in first-degree relatives compared with the normal population prevalence were reported also by Øyen et al. [22, 23] in a large national epidemiological cohort study regarding the period 1977–2005.

Lower ratios were found for the 2nd- and 3rd-degree relatives. Their study includes, however, also cases with associated chromosomal or extracardiac anomalies.

### 4.1. Concordance of Congenital Heart Lesions

Our results confirm a large variability in the recurrence of cardiac lesions, both in cases with multiple familial risk factors and in consecutive pregnancies, in agreement with other reports [8, 13]. In our series we found a complete concordance of recurrent CHD in 21.2% and a partial concordance in additional 20% of cases. A higher concordance was reported by Corone et al. [24] in 48% of affected first degree relatives and in 28% of affected second- and third-degree relatives and by Anderson [25] who found a complete concordance in 31% of affected siblings and in 46% of affected parents. Gill et al. [13] found an exact concordance in 37% and a group concordance in 44%; in families with two or more recurrences, the exact concordance was 55% and the exact concordance was high in AVSD 4/5 (80%) and laterality defects 7/11 (64%). 

Among discordant pairs of lesions, some occur more frequently and mainly within the group of septal defects (ASD, VSD, AVSD) or between TF, TGA, and VSD. We found also a case of recurrence of PS after the previous pulmonary atresia. Equally, the variability between different types of the left ventricular obstructive lesions has been reported in several studies [24, 26].

### 4.2. Recurrence of Specific Cardiac Lesions

#### 4.2.1. Atrioventricular Septal Defect

Familial occurrence of AVSD is reported both in the presence of euploidy and aneuploidy (trisomy 21), with a rate in siblings about 3-4% [2–5, 27, 28], the recurrent lesions being often concordant. The total recurrence rate in our study was 7.8%, 6.5% in siblings, all affected cases being nonsyndromic. In some families there were reported discordant lesions such as VSD, ASD, patent ductus arteriosus, and hypoplastic right ventricle with PS [29], similarly to our results. Vertical transmission found in selected pedigrees suggests an autosomal-dominant mechanism, with monogenic or oligogenic inheritance [8, 27]. Recurrence risk is about 10% for an affected parent, higher for mothers, 14% [28].

#### 4.2.2. Tetralogy of Fallot

Familial recurrence of CHD among patients with nonsyndromic TF was reported of 2.5–3% [1, 5] and 5.5% in our study. The model of transmission of TF is thought to be prevalently an autosomal recessive mechanism in selected families [30]. An etiologic relationship was reported for the conotruncal lesions, PS, and VSD [26].

*Transposition of great arteries* is generally considered to have a sporadic occurrence in families, with a risk of 2, 5-6, and 9%. In a more recent study [31] the recurrence in siblings was 1.8%, usually with a concordant lesion. This was not the case in our series, in which the total recurrence was 2.88% and 3.03% in siblings, with recurrence of VSD and PS.

*Corrected transposition of great arteries *had a higher recurrence of 5.2% in the study of Piacentini et al. [32] than in ours. In our population total recurrence was 1/15 = 6.7% and 1/10 = 10% in siblings, with an occurrence of a more complex form of the corrected transposition.

#### 4.2.3. Left Heart Lesions

J. J. Nora and A. H. Nora [6] indicate a recurrence in siblings and offspring of 2.2/3.9% for AS and 1.8/2.7% for aortic coarctation. Allan et al. [11] report a recurrence rate of 1/28 (3.6%) of aortic valve atresia and 1/15 ( 6.7%) of coarctation. In our population the recurrence for aortic stenosis was 4.3%, 2/27 (7.4%) when the mother was affected and 2/34 (5.9%) when the father was affected. Lower total recurrence was found for coarctation (2.24%) and was equally higher among affected mothers than fathers, 1/17 (5.9%) and 1/7 (14.3%), respectively. 

Obstructive lesions of the left outflow tract are presumed to be due to an altered embryonic blood flow and may occur in families with variable degrees of severity starting with bicuspid aortic valve, AS, coarctation of the aorta, and HLHS suggesting a genetic predisposition to the flow alterations [29, 33]. An autosomal recessive transmission has been suggested [34]. 

*Right-sided obstructive lesions and membranous ventricular septal defects* are also presumed to be due to altered flow and appear to be developmentally heterogeneous, occurring in familial combinations with conotruncal malformations, as expression of a “conotruncal susceptibility” or “forme frusta” manifestations of conotruncal abnormalities [24].

*Pulmonary atresia with intact ventricular septum and hypoplastic right heart* is considered to be a single-gene disorder [35] and in our study was found in 2 male siblings, with a high total recurrence rate of 2/14 (14.3%).

*Pulmonary stenosis* is known to be frequently associated with Noonan syndrome. An unusually high recurrence rate of 9.1% was reported in the Baltimore Washington Infants Study [19], while others reported a recurrence close to 2–2.7%. In our series, the total recurrence was 5.3% and in siblings 5.4%. The recurrent cases were heterogeneous (only one recurrence was concordant, other cases presented HLV, CoA, and partial anomalous venous drainage.). 

*Ventricular septal defect* has a recurrence rate between 1.7 and 4.2% in the literature [6]; its prevalence in the study of Moons et al. [17] was 2.72. Our total recurrence rate was 4.2%, with 1.59% in siblings and 7% in affected mothers and fathers. The recurrent lesions were either concordant or discordant (tricuspid atresia with VSD, TF, AVSD with hypoplastic right ventricle and VSD associated with ASD and PS and also TGA). 

*Familial atrial septal defect* is usually due to an autosomal dominant transmission. However, the genetics of ASD is considered to be heterogeneous. The ASD associated with conduction abnormalities seems to be a distinct entity with a different histopathologic origin, with possible relation to autoimmune and connective tissue disorders [19]. The recurrence of ASD was reported between 2.5 and 3.7% [6, 26, 27] and it was 4.1% in our entire series, but higher in fetuses from affected mothers and fathers (5.7% and 5%).

#### 4.2.4. Laterality Defects

Recurrence for the laterality defects have been reported to be between 2 and 4.75% [9, 25].

In our series, we have found a total recurrence of 1/22 (4.5%) in index cases with left and right isomerism and a concordant recurrence in siblings of 1/19 (5.3%) with left isomerism and AVSD, but with different associated intracardiac lesions. 

High relative risk ratio for heterotaxy was reported by Pradat and Øyen et al. [21, 22]. 

Out of 22 cases with dextrocardia, either with situs solitus and or inversus, recurrent lesions occurred in 2 cases (total recurrence 8.7% and rate in siblings 2/14–14.3%).

## 5. Conclusions

Our data demonstrate a higher recurrence rate of CHD than in previously published data, mainly thanks to an improved detection of even milder forms of CHD both in utero and after birth, and confirm the specific transmission of certain types of CHD in some families indicating a genetic component. Ongoing genetic and molecular studies suggest intriguing links between certain specific cardiac lesions in some familial pedigrees. The clinical significance of data regarding the recurrence of CHF is evident, particularly for preconception and prenatal counseling. 

The couple receiving prenatal counseling and surveillance should be informed of the impossibility of a total exclusion of the occurrence of some small defects and evolutive lesions as coarctation and that the global prognosis of the infant can be sometimes completed only after birth.

##  Disclosure

No funding was received for the manuscript.

## Supplementary Material

Supplementary Table S1: Concordance and discordance of CHD in index cases and affected fetuses. The Table shows in detail the type of CHD in index cases and in affected fetuses in cases with complete or partial concordance and discordance.

## Figures and Tables

**Table 1:** Recurrence of congenital heart defects in cases with single- and multiple-familial risk.

Index case with CHD	Total cases *n*.	Affected cases *n*.	Recurrence rate %
Single risk—total	1477	60	4.06
Previous child	818	29	3.5
Mother alone	250	13	5.2
Father alone	93	7	7.5
One relative II°-III°	316	11	3.5

Double cases risk	137	4	2.9
2 previous children	22	1	4.5
1 previous child + 1 relative	23	—	—
*Mother + another relative *	26	2	7.7
+ 1 previous child	8	1	11.1
+ father	3	1	6.7
+ 1 relative	15		
*Father + another relatives *	19	1	5.3
+ prev. child	12	1	8.3
+ 1 relative	7	—	—
2 relatives II°-III°	47	—	—

Multiple cases risk	20	1	5
2 prev children + 1 relative	2	—	—
Mother + prev. child or 2–4 relatives	5	1	20.0
Father + 2 relatives	3	—	—
3-4 relatives II°-III°	10	—	—

Total double + multiple risk	157	5	3.2

Total series	1634	65	3.98

**Table 2:** Recurrence of congenital heart defects in subgroups of CHD in index cases: total recurrence rate and recurrence rates in siblings, offspring, and distant relatives.

Index case diagnosis	Total index cases *n*.	Affected cases *n*.	Recurrence diagnosis	Recurrence rate Tot %	Recurrence rate in siblings %	Recurrence in offspring % M, F	Recurrence rate in Relatives %
*Anomalies of a-v connections*							
(i) UVH	65	1	1 HLV	1/65 = 1.5	1/52 = 1.9	M 0/1, F 0/1	0/11
(ii) HLV	81	1	1 HLV	1/81 = 1.23	1/80 = 1.25	—	0/1
(iii) TrAtr	37	—	—	—	—	—	—
(iv) AVSD	64	5	1 VSD; 1 AVSD+ HLV 2 AVSD incomplete; 1 cor triatriatum.dx.	5/64 = 7.8	3/46 = 6.5	M 1/4 = 25.0, F 0/1	1/13 = 7.7
(v) Ebstein/Dyspl Tr	21	2	1 LSVC; 1 CoA	2/21 = 9.5	2/15 = 13.3	M 0/1, F 0/5	—

*Conotruncal anomalies*							
(i) Tetralogy of Fallot	151	9	1 DORV; 1 ASD+PS; 2 VSD; 1 PLSVC; 1 PS; 1 TF+agen.PV, 1 Patr+IVS	8/151 = 5.3	2/73 = 2.7	M 1/26 = 3.8, F 2/11 = 18.2	3/41 = 7.3
(ii) PAtr + VSD	19	1	1 VSD	1/19 = 5.3	1/11 = 9.1	M 0/1	0/7
(iii) PAtr + IVS	14	2	2 PS	2/14 = 14.3	2/10 = 25	—	0/4
(iv) DORV	15	—	—	—	—	—	—
(v) Truncus	20	1	1 ASD	1/20 = 5	1/20 = 5	—	—
(vi) TGA	104	3	2 VSD; 1 PS	3/104 = 2.88	2/66 = 3.03	M 0/2	1/36 = 2.8
(vii) TGAc	15	1	1 TGAc + VSD + PAtr	1/15 = 6.7	1/10 = 10.0	M 0/1, F 0/2	0/2

*Shunts:*							
(i) VSD	239	10	1 TrAtr +TGA+VSD; 2 VSD; 1 TF; 2 AVSD 1 VSD + ASD + PS 2 ASD; 1 truncus	10/239 = 4.2	2/126 = 1.59	M 5/66 = 7.5, F 1/14 = 7.1	2/33 = 6.1
(ii) VSD + ASD	23	1	1 TGA	1/23 = 4.35	1/18=6.3	M 0/2, F 0/1	0/2
(iii) ASD	194	8	2 ASD; 3 VSD; 1 VSD + ASD; 1 AVSD + HLV + HAo; 1 ASD + PAPVD + agenesis right lung	8/194 = 4.1.	1/59 = 1.7	M 5/88 = 5.7, F1/20 = 5–0	1/27 = 3.7
(iv) Ductus Arteriosus	34	1	1 ASD	1/34 = 2.94	0/10	M 1/12 = 8.3, F 0/1	0/11

*Anomalies of semilunar valves/great arteries:*							
(i) CoA/Interr Ao arch	134	3	1 HLV; 1 ASD;1CoA	3/134 = 2.24	1/84 = 1.2	M 1/17 = 5.9, F 1/7 = 14.3	0/26
(ii) AS	115	5	1 VSD; 2 AS, 1 Ebstein; 1 TGA + CoA	5/115 = 4.3	0/28	M 2/27 = 7.4, F 2/34 = 5.9	1/26 = 3.8
(iii) PS	76	4	1 HLV; 1 PS; 1 CoA, 1 PAPVD	4/76 = 5.3	2/37 = 5.4	M 0/18, F 1/6 = 16.7	1/15 = 6.6

*Various:*							
(i) P/TAPVD	22	—	—	0/22-	0/12	M 0/2, F 0/3	0/5
(ii) Heterotaxy syndrome	22	1	1 AVSD + left isom;	1/22 = 4.5	1/19 = 5.3	—	0/3
(iii) Dextrocardia + svi, s.sol	23	2	1 HRV + PAtr; 1 AVSD + right isom	2/23 = 8.7	2/14 = 14.3	M 0/5, F 0/2	0/2

*Other (miscel.)*	41	1	1 VSD	1/41 = 2.4	0/17	M 0/7, F 0/6	1/11 = 9.1
*CHD undefined *	105	4	1 VSD; 2 CoA, 1 TGA	4/105 = 3.8	3/30 = 10	F 0/1	1/74 = 1.35

Total	1634	65		3.98			

*n*: number, CHD: congenital heart disease, c.: cases, F: father, M: mother, a-v: atrioventricular UVH: univentricular heart, AVSD: atrioventricular septal defect, HLV: hypoplastic left ventricle, HAo: hypoplastic aorta, HRV: hypoplastic right ventricle, TrAtr: tricuspid atresia, Dyspl Tr: dysplasia of the tricuspid valve, PV: pulmonary valve; PAtr: pulmonary atresia, IVS: intact ventricular septum, VSD: ventricular septal defect, DORV: double outlet right ventricle, TF: Tetralogy of Fallot, TGA: transposition of great arteries, TGAc: corrected transposition of great arteries, CoA: coarctation of aorta, Interr Ao: interrupted aortic arch, AS: aortic stenosis, PS: pulmonary stenosis, ASD: atrial septal defect ostium secundum; P/TAPVD: partial/total anomalous pulmonary venous drainage; PLSVC: persistent left superior vena cava; s.: syndrome; isom: isomerism; svi: situs viscerum inversus; s.sol: situs viscerum solitus, miscel.: miscellaneous.

**Table 3:** Relative risks ratios—comparison between our series and normal population (Moons et al.).

CHD	Our series (‰)	Moons et al. (‰)	RR	Confidence limits (95%)	*P*-value
HLV	1/1634 (.6 )	10/111225 (.09)	6.8	0.9–53.2	0.067
UHV	1/1634 (.6)	9/111225 (.08)	7.6	0.96–59.8	0.055
TF	9/1634 (5.5)	52/111225 (.5)	11.8	5.8–24.1	0.0001
PA + VSD	1/1634 (.6)	6/111225 (.05)	11.4	1.4–94.3	0.024
PAIVS	2/1634 (1.2)	6/111225 (.05)	29.7	4.6–112.6	0.0001
Truncus	1/1634 (.6)	7/111225 (.06)	13.7	1.7–111.1	0.0145
TGA	3/1634 (1.8)	29/111225 (.3)	7.05	2.1–23.2	0.0013
TGAc	1/1634 (.6 )	3/111225 (.03)	22.7	24–218	0.007
CoA	3/1634 (1.8)	46/111225 (.4)	4.5	1.4–14.3	0.0123
AVSD	5/1634 (3.1)	37/111225 (.3)	9.2	3.6–23.5	0.38
Ebstein	2/1634 (1.2)	3/111225 (.03)	63.8	10.7–382.4	<0.0001
PS	3/1634 (1.8)	88/111225 (.8)	2.3	0.7–7.3	0.007
As	5/1634 (3.1)	26/111225 (.2)	18.5	7.1–48.1	<0.0001
ASD	8/1634 (4.9)	162/111225 (1.5)	4.7	2.3–9.7	<0.0001
VSD	10/1634 (6.1)	303/111225 (2.7)	2.3	1.9–4.2	0.0008
T/PAPVD	1/1634 (.6)	28/111225 (.3)	3.4	0.46–25.1	0.2277

CHD: congenital heart disease, HLV: hypoplastic left ventricle, UVH: univentricular heart, TF: Tetralogy of Fallot, PAtr: pulmonary atresia, PAIVS: pulmonary atresia + intact ventricular septum, TGA: transposition of great arteries, TGAc: corrected transposition of great arteries, CoA: coarctation of aorta, AVSD: atrioventricular septal defect, PS: pulmonary stenosis, AS: aortic stenosis, ASD: atrial septal defect ostium secundum, VSD: ventricular septal defect, T/PAPVD: total/partial anomalous pulmonary venous drainage.

## Abbreviations

CHD: Congenital heart disease
HLHS, HLV: Hypoplastic left heart syndrome, hypoplastic left ventricle
AVSD: Atrioventricular septal defect
VSD: Ventricular septal defect
ASD: Atrial septal defect ostium secundum
TGA: Transposition of great arteries
PS: Pulmonary stenosis
AS: Aortic stenosis
CoA: Coarctation of the aorta
TF: Tetralogy of Fallot
TGA: Transposition of great arteries
TGAc: Corrected transposition.
