# Supplementary material for: Recurrence of Congenital Heart Disease in Cases with Familial Risk Screened Prenatally by Echocardiography
Source: J Pregnancy. 2011 Oct 1;2011:368067. doi: 10.1155/2011/368067 (PMC3184425; doi:10.1155/2011/368067)
Supplement: Supplementary file 1 — Supplementary Table S1: Concordance and discordance of CHD in index cases and affected fetuses. The Table shows in detail the type of CHD in index cases and in affected fetuses in cases with complete or partial concordance and discordance. [file 368067.f1.doc]

**Table n. S1: Concordance and discordance of CHD in index cases and affected fetuses**

| Type of recurrence | N. | CHD in index case CHD in affected fetus |
| --- | --- | --- |
| Complete concordance | 14 | ASD in mother idem  VSD in mother idem  VSD in previous child idem  AVSD in previous child idem  PS in previous child idem  AS bicuspid aorta in father idem  AS in previous child idem  CoA in mother idem  HLV in previous child idem  AVSD+ HLV in previous child idem  T.Fallot in grandmother T.Fallot + agenesis PV  TGAc+ Patr+ VSD in previouschild idem  Dxc, svi, HRV previous child idem  **AVSD incomplete in mother+ 3 relatives idem |
| Partial concordance –  Milder or similar entity or within group | 8 | L isom, AVSD, HAo in previous child. L isom, AVSD,TGA, PS  ASD in II° relative VSD  ASD s.ven.in mother VSD  VSD in mother ASD  VSD in father ASD PDA in mother ASD  T.Fallot in father DORV  **ASD and VSD in mother and previouschild ASD |
| Partial concordance –  more complex, or within group | 5 | ASD in mother VSD+ASD  ASD in mother ASD+ PDA+ PAPVD+ agenesis Rt. lung  VSD in previous child VSD+ ASD II+ PS  VSD in mother - AVSD  CoA in father HLV |
| Discordance  more complex | 10 | ASD in previous child AVSD+ HLV  VSD in mother AVSD + HRV  VSD in II° relative TrAtr.  VSD in II° relative truncus  VSD+ ASD in previous child TGA  AS in mother Ebstein  TGA in II° relative Patr+ HRV+ displ.Tr.  PS in II° relative HLV  UVH+ PS in previous child HLV  ****VSD in mother + previous child T.Fallot |
| Discordance – milder CHD | 19 | T.Fallot in father VSD  T.Fallot in previous child ASD + PS  T.Fallot in previous child VSD  T.Fallot in grandmother PSI-dyspl.PV  T. Fallot in mother PLSVC  TGA in previous child VSD  TGA in previous child VSD  PAtr + IVS in previous child PS  PAtr + IVS in previouschild PS  PAtr + VSD in previous child VSD  AVSD in previouschild VSD  AVSD in III° relative PS+ HRV+ cor tr.dx  AS in father VSD  CoA in previouschild ASD  Ebstein + CoA in prev child CoA  Ebstein + PLSVC in previouschild PLSVC  Cong. mitral dysplasia in grandfather VSD  **Truncus and HAo in 2 previouschildren ASD  **PS+ASD and PS+ASD+CMP in father  and previouschild VSD |
| Discordance –  similar gravity | 5 | AS + HCMP in mother TGA+CoA  PS in father CoA  PS in previouschild PAPVD cor tr.  T.Fallot in grandmother PAtr + IVS  Dxc, TGAc, PAtr in previouschild Rt.isom, AVSD, PS |
| Unknown | 4 | Undefined CHD in previouschild VSD  Undefined CHD in previouschild CoA  Undefined CHD in previouschild CoA Undefined CHD in II* relative TGA |

**Legend**: N- number, CHD – congenital heart disease, previous- previous, ASD- atrial septal defect ostium secundum VSD – ventricular septal defect, AVSD – atrioventricular septal defect, PS – pulmonary stenosis, PSI – pulmonary stenoinsufficiency, , AS – aortic stenosis, CoA – coarctation of aorta, HLV- hypoplastic left ventricle, HAo – hypoplastic aorta, HRV – hypoplastic right ventricle, T.Fallot- Tetralogy of Fallot, UVH – univentricular heart, TrAtr – tricuspid atresia, Dyspl. Tr – dysplasia of the tricuspid valve, PV –pulmonary valve; PAtr – pulmonary atresia, IVS - intact ventricular septum, DORV – double outlet right venricle, TGA – transposition of great arteries, TGAc – corrected transposition of great arteries, Interr Ao – interrupted aortic arch, PS – pulmonary stenosis; PAPVD – partial anomalous pulmonary venous drainage cong,- congenital, PLSVC – persistent left superior vena cava; CMP- cardiomyopathy, HCMP- hypertrofic cardiomyopathy, s. – syndrome; , Rt- right, L- left, isom – isomerism; svi – situs viscerum inversus; s.sol – situs viscerum solitus, dxc- dextrocardia, s.ven- sinus venosus, incompl- incomplete, cor tr. dx- cor triatriatum dexter
